# A Case of Possible Monosodium Glutamate-Dependent, Exercise-Induced Anaphylaxis

**DOI:** 10.7759/cureus.5345

**Published:** 2019-08-08

**Authors:** Julian S Trent, Stephanie Tassin

**Affiliations:** 1 Emergency Medicine, San Antonio Military Medical Center, San Antonio, USA

**Keywords:** exercise-induced, food-dependent, anaphylaxis

## Abstract

A 24-year-old Asian-American male presented to the Emergency Department with his third episode of anaphylaxis in a one-year period. Based on the clinical history provided by the patient regarding each of these episodes, the trigger for the patient’s anaphylaxis appears to be the consumption of monosodium glutamate followed by physical exertion within the subsequent two to three hours. The case presented here represents a rare, but life-threatening, disorder. Identification of the triggers is important so that steps can be taken to prevent recurrence.

## Introduction

Exercise-induced anaphylaxis is an under-identified variant of anaphylaxis, with an estimated prevalence of up to 15% of cases of anaphylaxis [1]. A further subset of patients with exercise-induced anaphylaxis includes individuals whose anaphylaxis is dependent on the ingestion of certain food products prior to exercise. Unlike other cases of anaphylaxis where exposure to the trigger and the development of symptoms occurs in a close temporal relationship, the development of symptoms in food-dependent, exercise-induced anaphylaxis (FDEIA) can occur hours after ingestion of the trigger.

## Case presentation

The patient was jogging when he developed an urticarial rash of the trunk and bilateral upper and lower extremities, nausea, abdominal pain, angioedema of the tongue and face, and cardiovascular instability. A total of 0.3 mL of 1 mg/mL epinephrine was administered in the left lateral thigh via the patient’s commercial epinephrine auto-injector. Emergency Medical Services were called to transport the patient to the Emergency Department where intravenous diphenhydramine, methylprednisolone, and ranitidine were administered. Over the course of a three-hour observation period, the patient’s symptoms completely resolved and he was discharged. This episode of anaphylaxis, along with the previous two episodes the patient suffered, occurred following the consumption of monosodium glutamate-containing products prior to exercise. Independent of exercise, the patient is able to tolerate monosodium glutamate without adverse reactions, and the patient is able to tolerate strenuous physical activity without complications in the absence of exposure to monosodium glutamate in the preceding few hours.

## Discussion

In any case of suspected food-dependent, exercise-induced anaphylaxis, the goals of identifying the offending agent are to prevent recurrence of the allergic reaction, prevent an unnecessarily restricted diet, and to allow the individual to continue participation in strenuous physical activity. In cases of successful identification of the allergen, individuals with this rare disease are afforded the opportunity to continue their regular diet and exercise regimens with simple precautions, namely avoiding the trigger for four to five hours prior to exercise [2]. Unlike other cases of food-dependent anaphylaxis in which any exposure to the allergen can produce life-threatening symptoms, FDEIA requires exercise. Additionally, in some cases, certain environmental factors such as ambient temperature, humidity, or the recent ingestion of non-steroidal anti-inflammatory drugs (NSAIDs) are necessary for the development of symptoms [2]. The recommended prevention strategy is to avoid the allergen four to five hours pre-exercise, and at least one hour post-exercise [3].

While the pathophysiology for FDEIA is not completely understood, de-granulation of mast cells appears to be implicated in the disease process. It is theorized that exercise releases co-factors necessary for the sensitization of the mast cells, lowering the threshold for degranulation and the release of histamine and other vasoactive peptides [4]. Moreover, exercise may increase the presence of the allergen in the blood stream by increasing absorption from the gastrointestinal tract, leading to an increase in the number of immunoglobulin E antibodies being bound to the antigen [5]. In certain cases, the threshold for mast cell degranulation requires contributory environmental conditions; most commonly cold weather, recent ingestion of NSAIDs, high humidity, viral infection, or fatigue [4]. The treatment for FDEIA, like in other causes of anaphylaxis, is primarily epinephrine. Anti-histamines and steroids are also frequently used as adjuncts for symptomatic management [3].

The most commonly implicated allergens in FDEIA are wheat and crustaceans [4-5]. However, other allergens including soybeans, chickpeas, mustard, and apple have been reported [6-8]. This case represents a novel food product that appears to be required for the development of exercise-induced anaphylaxis, monosodium glutamate, based on the clinical history provided by the patient. One limitation of this case is the lack of laboratory data to support the diagnosis. The severity of the reaction prohibits traditional provocative testing with an exercise challenge, and serum-specific immunoglobulin E testing and skin prick testing are often unreliable [5, 9]. In this case, it is suspected that monosodium glutamate represents a cofactor further sensitizing the individual to the development of anaphylaxis, similar to other cases in which ambient temperature, accompanying ingestion of NSAIDs, or the presence of a viral infection is required to develop a reaction to an antigen [9].

## Conclusions

FDEIA is a rare, but important disease that can be difficult to diagnose with traditional allergy testing. The identification of the offending agents is crucial to preventing unnecessary limitations to diet and exercise. The disease process may be more complicated than previously understood requiring not only the presence of an allergen and exercise but other environmental factors or exposures. 
